# Strategies to Increase Drug Penetration in Solid Tumors

**DOI:** 10.3389/fonc.2013.00193

**Published:** 2013-07-26

**Authors:** Il-Kyu Choi, Robert Strauss, Maximilian Richter, Chae-Ok Yun, André Lieber

**Affiliations:** ^1^Department of Bioengineering, College of Engineering, Hanyang University, Seoul, South Korea; ^2^Genome Integrity Unit, Danish Cancer Society Research Center, Copenhagen, Denmark; ^3^Department of Medicine, University of Washington, Seattle, WA, USA

**Keywords:** epithelial junctions, tumor stroma, extracellular matrix, relaxin, junction opener, tumor-associated macrophages

## Abstract

Despite significant improvement in modalities for treatment of cancer that led to a longer survival period, the death rate of patients with solid tumors has not changed during the last decades. Emerging studies have identified several physical barriers that limit the therapeutic efficacy of cancer therapeutic agents such as monoclonal antibodies, chemotherapeutic agents, anti-tumor immune cells, and gene therapeutics. Most solid tumors are of epithelial origin and, although malignant cells are de-differentiated, they maintain intercellular junctions, a key feature of epithelial cells, both in the primary tumor as well as in metastatic lesions. Furthermore, nests of malignant epithelial tumor cells are shielded by layers of extracellular matrix (ECM) proteins (e.g., collagen, elastin, fibronectin, laminin) whereby tumor vasculature rarely penetrates into the tumor nests. In this chapter, we will review potential strategies to modulate the ECM and epithelial junctions to enhance the intratumoral diffusion and/or to remove physical masking of target receptors on malignant cells. We will focus on peptides that bind to the junction protein desmoglein 2 and trigger intracellular signaling, resulting in the transient opening of intercellular junctions. Intravenous injection of these junction openers increased the efficacy and safety of therapies with monoclonal antibodies, chemotherapeutics, and T cells in mouse tumor models and was safe in non-human primates. Furthermore, we will summarize approaches to transiently degrade ECM proteins or downregulate their expression. Among these approaches is the intratumoral expression of relaxin or decorin after adenovirus- or stem cell-mediated gene transfer. We will provide examples that relaxin-based approaches increase the anti-tumor efficacy of oncolytic viruses, monoclonal antibodies, and T cells.

## Tumor Microenvironment

### Tumor stroma

**Figure 1:**
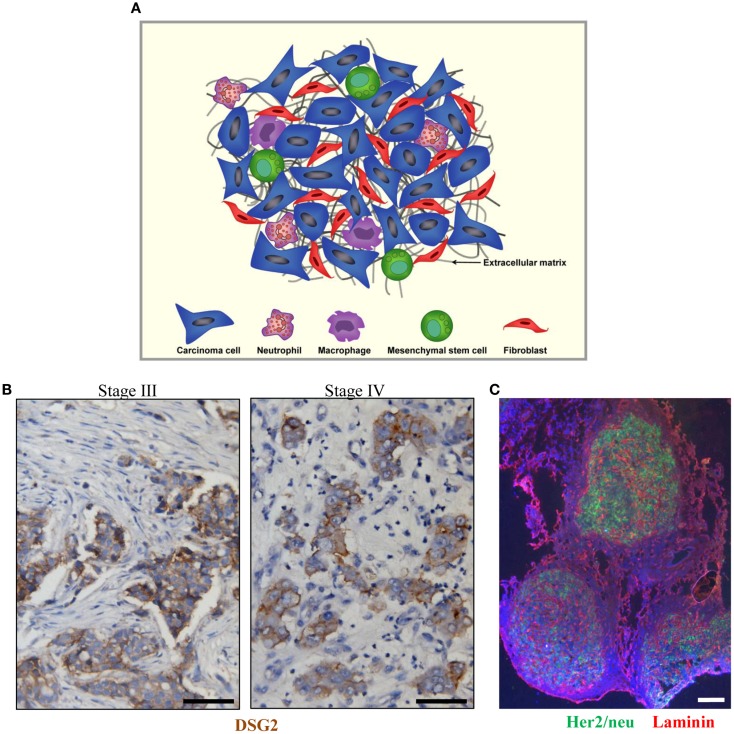
**Tumor stroma**. **(A)** Schematic representation of tumor stroma components. The tumor stroma is composed of stromal cells (fibroblasts, macrophages, neutrophils, and mesenchymal stem cells) as well as extracellular matrix. **(B)** Sections from breast cancer patient biopsies (stage III and IV). DSG2 staining appears in brown. Malignant cells are DSG2-positive and form nests that are surrounded by tumor stroma containing DSG2-negative stroma cells such as fibroblasts. **(C)** Immunofluorescence analysis for Her2/neu (green) and the stroma protein laminin (red) on a breast cancer section. The scale bar is 20 μm.

Tumors are heterogeneous cellular entities in which progression depends on the crosstalk between the genetically abnormal cells (the epithelial parenchyma of carcinomas) and the tumor stroma (the supportive framework of a tumor tissue). This tumor stroma is basically composed of the non-malignant cells (stromal cells) of the tumor such as cancer-associated fibroblasts (CAFs), immune cells [tumor-associated macrophages (TAMs) and tumor-associated neutrophils (TANs)], and mesenchymal stem cells as well as the extracellular matrix (ECM) consisting of fibrous structural proteins (collagen and elastin), fibrous adhesive proteins (fibronectin and laminin), and proteoglycans (1–3) (Figure 1A). In most solid tumors derived from epithelial tissues, nests of malignant tumor cells are linked through junction proteins such as E-cadherin, claudins, and desmoglein 2 (DSG2). Tumor nests are surrounded by tumor stroma (Figures 1B,C). The stroma is indispensable for normal tissue development and homeostasis, since it has a vital role in regulating behavior of cells residing in the local milieu (4–7). Likewise, various components of the tumor stroma create a niche favoring seeding of metastatic tumor cells. More importantly, tumor stroma mediates the resistance to cancer therapeutic agents (8–10). Tumor stroma contributes in at least two critical ways to drug resistance: (i) by creating a physical barrier formed by stroma proteins that prevents intratumoral drug penetration and direct contact between drugs/tumor-infiltrating immune effector cells and their target receptors on malignant cells and (ii) by production of cytokines and chemokines that trigger the synthesis of stroma proteins, block activation of immune cells, or attract/activate immuno-suppressive cells such as regulatory T cells (T_regs_).

It is well established that stroma that is associated with normal tissue development and homeostasis is strikingly distinct from that associated with carcinomas (1). Specifically, the composition of tumor-derived ECM is different from normal ECM (11). Excess ECM production or reduced ECM turnover are noticeable in the majority of tumors (12, 13). Various collagens (e.g., collagen type I, II, III, V, and IX), fibronectin, tenasin C, and proteoglycans exhibit increased accumulation and generate a dense network in tumor tissues (14–17). Excessive deposition of ECM components decreases the distance between neighboring ECM components and diminishes the pore size of the tumor matrix. This adds diffusional impediment to macromolecules (IgG, IgM, and dextran 2,000,000 MW) in tumors (18). A strong inverse correlation between tumor ECM content and tumor penetration of cancer drugs has been demonstrated for therapeutic agents such as anti-tumor immune cells, therapeutic viruses, chemotherapeutic agents, monoclonal antibodies, immunotoxins, interferons, and complement (18–24). Due to increased ECM deposition, tumor tissue commonly exhibits increased stiffness compared to normal tissue. For breast cancer, tumor tissue was found to be 10 times stiffer than normal breast tissue (26, 27). The elevated ECM stiffness progressively increases interstitial fluid pressure (IFP) which thereafter interferes with effective spread of anti-cancer therapeutics within the solid tumor (28–30). In summary, the deregulation and disorganization of the tumor stroma alter the composition, structure, and stiffness of the ECM, leading to limited penetration and dissemination of therapeutic agents within solid tumors. Killing the genetically stable tumor stroma cells has definitive advantages over targeting the malignant cells, i.e., cells that are genetically unstable and heterogeneous and represent a moving target for therapies.

### Tumor stromal cells

The major contributors of abnormal ECM in solid tumors are stromal cells such as cancer-associated fibroblasts (CAFs), TAMs, and TANs (3, 13, 31). These stromal cells display sustained synthesis and secretion of connective tissue components, growth factors, and cytokines, which promote the ability of malignant cells to proliferate, invade, and metastasize (32, 33). Thus targeting the tumor stromal cells is considered a promising approach to the treatment of cancer.

#### Cancer-associated fibroblasts

Cancer-associated fibroblasts are the predominant cell type in the tumor-associated stroma. Their numbers are elevated in tumor stroma compared to stroma found in healthy tissue (34, 35). In many carcinomas, the fraction of CAFs is even greater than the fraction of malignant cells (32). In the tumor microenvironment, tumor and stromal cells upregulate various profibrotic growth factors such as transforming growth factor-β (TGF-β), platelet-derived growth factor (PDGF), and basic fibroblast growth factor (bFGF), all of which are main mediators for the transdifferentiation of stromal fibroblasts into CAFs (36, 37). CAFs are phenotypically and functionally distinct from normal stromal fibroblasts (38). CAFs are large spindle-shaped mesenchymal cells that share morphological characteristics of both smooth-muscle cells and fibroblasts (39). Metabolically, CAFs are perpetually activated, proliferate faster, and accumulate greater amounts of ECM constituents than fibroblasts in normal tissues (2, 32, 40). In addition to creating tumor-derived ECM, CAFs have an impact on cancer cell proliferation, invasion, and metastasis through secretion of different growth factors [epidermal growth factor (EGF), FGF, hepatocyte growth factor (HGF), and insulin-like growth factor-1 (IGF-1) (2, 3, 31, 34, 41–44)]. CAFs are also involved in the activation of angiogenic programs as well as the recruitment of inflammatory cells (45). Local expression of vascular endothelial growth factor (VEGF) or monocyte chemoattractant protein-1 (MCP-1) by CAFs stimulates angiogenesis and the recruitment of pro-tumor myeloid cells.

#### TAMs and TANs

As another major component in the tumor stroma, TAMs have emerged as a significant player in the stromal compartment of virtually all types of carcinoma (46). While type M1 macrophages are antigen-presenting cells that incite T cells to mount immune responses, TAMs are M2-type macrophages and tumor promoting. Tumor cells, among other cytokines, produce MCP-1 and colony stimulating factor-1 (CSF-1) which participates in mobilization of TAM-progenitors from the bone marrow and homing to tumor stroma. Homing of TAMs to tumors is also supported by the specific architecture of tumor blood vessels which promote efficient trafficking of blood cells. There is convincing evidence that the extent of MCP-1 expression in human cancers correlated with both TAM infiltration and tumor malignancy (47–53). TAMs contribute to tumor-associated alteration in the ECM by releasing profibrotic growth factors, which then act in an autocrine and/or paracrine manner to differentiate normal stromal fibroblasts into CAFs (33, 37, 46). TAMs also produce growth factors (EGF, HGF, bFGF, and VEGF), cytokines [IL-1, IL-8, and tumor necrosis factor-α (TNFα)], and enzymes [MMP-2, MMP-7, MMP-9, MMP-12, and cyclooxygenase-2 (COX-2)] (46, 54). Additionally, TAMs can suppress anti-tumor immune responses. For example, TAMs secret a distinctive set of cytokines (IL-10 and TGF-β) as well as chemokines [chemokine (C–C motif) ligand (CCL)17, CCL22, and CCL24] favoring recruitment of T_regs_ and generation of an immune suppressive microenvironment (55–57). As outlined in Section “Epithelial-to-Mesenchymal Transition and Mesenchymal-to-Epithelial Transition in Cancer,” TAMs also promote epithelial-to-mesenchymal transition (EMT) via TGF-β (58) and regulate cancer stem cell (CSC) activities (59) in solid tumors.

Tumor-associated neutrophils comprise another prominent portion of the immune cell infiltrates observed in a wide variety of murine models and human cancers (60–63). Similar to TAMs, products secreted from neutrophils, including reactive oxygen species, cytokine (IL-8), growth factors (VEGF and HGF), and proteinases [arginase (ARG 1), MMP-2, MMP-8, MMP-9, and MMP-13], have defined and specific roles in both regulating tumor cell proliferation, angiogenesis, and metastasis and suppressing the anti-tumor immune response (64).

### Tumor vascularization

#### Transendothelial transport

Once systemically administered drugs reach the tumor sites, they have to exit the tumor vasculature and translocate through the interstitial space in order to reach their target cells. The endothelial cell layer, lining the blood vessels, is thought to present a barrier to macromolecular drugs (20). Transendothelial transport of macromolecular drugs involves a phenomenon known as the enhanced permeability and retention (EPR) effect in solid tumors. The EPR effect is observed for intravenously administered macromolecular anti-cancer drugs that escape renal clearance, due to their large molecular size (10–500 nm). They are mostly unable to pass the tight endothelial junctions of normal blood vessels, but can extravasate and then become trapped in the tumor vicinity (65). Unlike normal tissues that feature an organized vascular network, the blood vessel system in solid tumors is rather chaotic. The endothelial cell layers are poorly aligned (66) and elevated levels of vascular permeability factors generate “leaky” capillaries (65). It is therefore thought that transendothelial transport is not a critical limiting obstacle for large sized drugs.

#### Intratumoral pressure

Rapid tumor cell proliferation and weakly developed lymphatics cause high IFP (67, 68) and blood vessel remodeling by intussusception (69) or compression (70). Additionally, the increased hydraulic conductivity of “leaky” capillaries can further increase the IFP in tumors (71). Together, this leads to an imbalance in blood flow and nutrient supply within the tumor microenvironment. The uniformly high IFP in the center of solid tumors drops toward the periphery (72), which could negatively affect drug extravasations in the high-pressure regions. Cells that are distant to blood vessels (100–200 μm) and located in high-pressure regions subsequently constitute large areas of hypoxic, necrotic, or semi-necrotic tissue. This exacerbates the tendency of tumor cells to overproduce and release lactic acids within these regions, which results in acidosis (73). Moreover, the vascular surface area per unit tissue weight is decreasing with tumor growth, which further limits transvascular exchange for large tumors when compared to small tumors (74, 75). In contrast, cells situated in the invasive front benefit from the enhanced vascular permeability that supplies adequate amounts of macromolecules for rapid tumor growth (76). Furthermore, the blood flow rates in non-necrotic regions can be substantially higher than in the surrounding normal tissue (77). It is therefore expected that the uptake of drugs in solid tumors is heterogeneous and the general distribution might decrease with increasing tumor weight. It is thought that induction of massive cell death by chemo- and radio-therapy can lower the IFP in tumors (78). The application of chemotherapy to lower the IFP is also used in approaches to “normalize” the tumor vasculature. Anti-angiogenic drugs are thought to compensate for the pro-angiogenic factors that are extensively produced in the tumor in order to eliminate “leaky” blood vessels. Ideally, this would lead to a more organized blood vessel system that features more functional and more uniformly perfused capillaries within solid tumors. On the other hand, this would also inhibit the extravasation of large drugs.

#### Hypoxia

Two major consequences of abnormal microcirculation in solid tumors are hypoxia and low extracellular pH. Hypoxia or oxygen deprivation is a key factor in tumor progression and resistance to therapy. The most important regulatory factor of the hypoxia-signaling pathway activity in cells is hypoxia-inducible transcription factor 1 (HIF-1α). Under hypoxic condition, tumors produce a number of chemokines that attract and differentiate CAFs, TAMs, and TANs. Approaches that reduced intratumoral hypoxia therefore block pro-tumoral functions of these cells, including the production of stroma proteins. It is therefore thought that hypoxia targeting strategies improve the intratumoral penetration of drugs (79).

## Epithelial Phenotype of Solid Tumors

### Epithelial junctions

**Figure 2:**
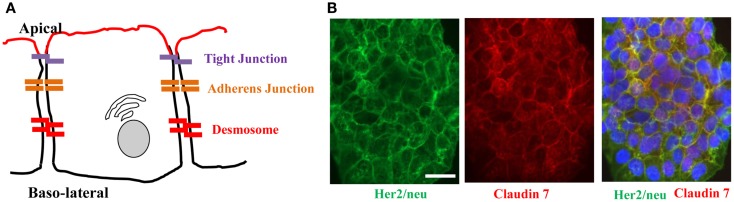
**Architecture of epithelial cells**. **(A)** Adjacent epithelial cells maintain several intercellular junctions and an apical-basal polarity. Tight junctions seal the paracellular space close to the apical side. Initial cell contact is initiated by cadherins in the adherens junction complex that is situated underneath tight junctions. Adherens junction complexes encircle cells as an adherens belt, which connects to the F-actin cytoskeleton. Desmosomes are spot-like adhesions randomly arranged on lateral sides of plasma membranes. A key desmosomal junction protein is desmoglein 2 (DSG2). **(B)** Target receptors for cancer therapy are often trapped in epithelial junctions. Shown are breast cancer cells stained for Her2/neu (the target receptor for trastuzumab/Herceptin™) and the junction protein claudin 7. The merged image shows colocalization of both proteins in junctions between cells.

About 90% of solid tumors are of epithelial origin, often featuring a stratified epithelium characterized by multilayered cells with three-dimensional intercellular junctions. This is in contrast to monolayered epithelial cells lining epithelial tracts (e.g., airway, gastrointestinal, and urinary tracts), epithelial ducts (e.g., bile and pancreatic ducts), or cavities (e.g., brain ventricles), which possess an apical-basal polarization of their cell membranes and cytoskeleton. The epithelial phenotype is generally defined by tight and adherence junctions that seal the paracellular space between adjacent cells and thereby providing a barrier that restricts passing of ions and macromolecules (Figure 2A) (80).

#### Tight junctions (zonula occludens)

Tight junctions play a key role in the formation of epithelial sheets. Strictly linked to tight junctions is a barrier function within a sheet of cells that restricts ions and small molecules to pass through the paracellular space between two adjacent epithelial cells (80). Additionally, tight junctions function as a “fence” that separates the apical and basal membrane compartments in an individual cell (81). Importantly, the tight junction strands on one cell are associated laterally to tight junction strands of opposing membranes on neighboring cells (82). The permselective barrier function is based on occludins and claudins, two types of transmembrane proteins that have been identified among more than 40 proteins within tight junctions (81, 83). Other tight junction transmembrane proteins comprise the singlespan junctional adhesion molecules (JAMs) or coxsackie and adenovirus receptor (CAR) (83–86) and the lately identified tetraspan tricellulin, which is enriched in areas where three cells meet (86).

#### Adherens junctions (zonula adherens)

The major transmembrane proteins of adherens junctions are classical cadherins, such as epithelial cadherin (E-cadherin). Members of this protein superfamily promote homophilic intercellular adhesion in a Ca^2+^-dependent manner. Their cytoplasmic domain binds cytosolic catenins that link the cadherin/catenin complex to the actin cytoskeleton. The formation of adherens junctions consequently leads to the assembly of tight junctions, but E-cadherin is dispensable for tight junction maintenance (87). A central role in maintenance and initiation of an epithelial phenotype is attributed to E-cadherin, the core protein of adherens junctions. In addition to its homophilic intercellular adhesive features, the extracellular E-cadherin domain functions as a direct repressor of receptor tyrosine kinase (RTK) signaling via blockage of FGF or EGF ligand binding stimulation (88, 89). The cytoplasmic part of E-cadherin connects to the actin cytoskeleton and also influences a number of signaling pathways via direct binding to p120- and β-catenin. When adjunct to E-cadherin at the membrane, β-catenin inhibits cell growth (90), whereas its translocation to the nucleus activates canonical Wnt signaling (91). Similarly, p120-catenin stabilizes E-cadherin at the membrane, while blocking NF-κB and Ras-MAPK signaling (92, 93).

#### Desmosomal junctions/desmosomes (macula adherens)

Desmosomes are molecular complexes of cell adhesion proteins and linking proteins that attach the cell surface adhesion proteins to intracellular keratin cytoskeletal filaments. The cell adhesion proteins of the desmosome, desmoglein, and desmocollin, are members of the cadherin family of cell adhesion molecules. They are transmembrane proteins that bridge the space between adjacent epithelial cells by way of homophilic binding of their extracellular domains to other desmosomal cadherins on the adjacent cell. Both have five extracellular domains, and have calcium-binding motifs. One of these junction proteins, DSG2, is upregulated in malignant cells (94, 95). DSG2 contains four extracellular cadherin domains (ECDs; ECD1–ECD4), which link neighboring cells to each other through homodimers. ECDs are linked via an extracellular anchor and membrane-spanning domain to the intracellular anchor and intracellular cadherin-typical sequence (ICS) motifs. The role of the conserved ICS is not known, although consensus sites for protein kinase C phosphorylation and a caspase-3 cleavage site have been identified and could contribute to signaling (96). Desmosomes probably do not directly regulate paracellular permeability, but they seem to do this indirectly by altering the structure and the stability of tight junctions (97).

### Block of intratumoral diffusion of macromolecules

One of the key features of epithelial tumors is the presence of intercellular junctions, which link cells to one another, and act as barriers to the penetration of molecules with a molecular weight of>400 Da (98–100). Most of commonly used chemotherapy drugs are either nanoparticle-based or encapsulated into liposomes with diameter greater than 100 nm. For example, nanoparticle albumin bound paclitaxel/nab-paclitaxel/Abraxane™ has an effective diameter of 130 nm and liposomal doxorubicin/Doxil™ has a size of 90 nm. Even non-encapsulated chemotherapy drugs have a molecular weight of greater 400 Da (for example: paclitaxel/Taxol™: MW 856.9 Da or irinotecan/Camptosar™: MW 586.7 Da). Several studies have shown that upregulation of epithelial junction proteins correlated with increased resistance to therapy, including therapy with the two major classes of cancer drugs – monoclonal antibodies and chemotherapeutics (101–103). It is thought that the epithelial phenotype of cancer cells and their ability to form physical barriers protect the tumor cells from attacks by the host-immune system or from elimination by cancer therapeutics (104).

### Inaccessibility of therapy target receptors

Receptors for therapeutic antibodies are often localized at the baso-lateral membrane of epithelial cells. This includes Her2/neu (105, 106) and EGFR (107, 108). In our studies on epithelial tumors we found that target receptors are trapped in intercellular junctions (109). For example, Her2/*neu*, the receptor for the widely used monoclonal antibody Herceptin (trastuzumab) co-stained with the tight junction proteins claudin 7 (Figure 2B).

### Epithelial-to-mesenchymal transition and mesenchymal-to-epithelial transition in cancer

Epithelial-to-mesenchymal transition and mesenchymal-to-epithelial transition (MET) are important mechanisms that drive tumor progression and therapy resistance, and indirectly affect intratumoral drug penetration. EMT and MET have been accredited important roles in embryogenic development, tissue regeneration, cancer progression, and recently also the induction and maintenance of stem cell properties (110). Importantly, the phenotypic switches between epithelial and mesenchymal phenotypes are not irreversible, as they occur several times during formation of the complex three-dimensional structure of internal organs. In contrast to epithelial cells, mesenchymal cells exhibit an irregular shape, which is based on unpolarized cytoskeletons and membranes. Further mesenchymal traits include the deposition of ECM components, increased motility, invasiveness, as well as elevated resistance to apoptosis and anoikis (110). EMT engages a series of events involving inter- and intra-cellular changes in affected cells. Importantly, not all of which have to occur during the transdifferentiation process. Often cells remain in stages referred to as an “incomplete” EMT, suggesting a spectrum of intermediate stages rather than a strict lineage switch (104). Examples of such epithelial/mesenchymal (E/M) hybrid cells have been reported for multiple tissues including the ovarian surface epithelium (111), ovarian cancer (25), cells within the invasive front of colon (112) and breast cancer (113), as well as in normal epidermal tissue during wound healing (114).

#### EMT and induction of tumor ECM proteins

During progression toward metastatic disease carcinoma cells engage EMT. The EMT program can be activated by a multitude of factors secreted by tumor stroma cells, which triggers a complex signaling network including TGF-β, Wnt, HGF, EGF, and PDGF pathways (115). The morphological changes that occur during EMT are a consequence of diverse molecular mechanisms that contribute to the acquisition of mesenchymal features. A central event during EMT is the functional loss of E-cadherin. Subsequent breakdown of intercellular epithelial junctions plays a major role in cancer progression, where E-cadherin therefore acts as a repressor of invasion (116). Accordingly, the reduced expression of this major regulator of the epithelial phenotype is associated with poor prognosis in several cancers (117). The loss of E-cadherin and several other epithelial genes including multiple members of the claudin family as well as occludin is mainly regulated via transcriptional repression by EMT inducers that include transcription factors Snail, ZEB, Twist, FOXC2, and E47 (118–126). Notably, these EMT inducers also act as positive regulators of gene expression for several mesenchymal genes (127, 128). A consequent event in EMT is the change from E-cadherin to N-cadherin (129). The importance of this cadherin switch is highlighted by the fact that homophilic intercellular junctions formed by N-cadherin are less resistant to rupture under physiological stress conditions, when compared with E-cadherin (130). Additionally, a shift from several keratins (-8, -9, and -18) to vimentin occurs, resulting in a more flexible cytoskeleton (131, 132). Concomitantly with the acquisition of such mesenchymal features, the expression of several ECM proteins is induced. Fibronectin, collagen precursors, laminin, and vitronectin are all reported to be elevated in mesenchymal cells (133). These and other proteins, including Src kinase, integrin-linked kinase, integrin β-5, and MMPs, are upregulated during EMT, have an impact on cytoskeletal remodeling, and promote cell motility (104).

#### MET and cancer stem cells

Successful EMT induction ultimately enables cancer cells to leave the primary tumor, enter the bloodstream, and attach to distant organ sites in order to build metastases. The endpoint of this process however, involves the reversed process (MET), where cells that underwent EMT regain epithelial properties and form tumors that histopathologically resemble the primary cancer (110, 134). Although the underlying mechanisms are currently unknown, it is likely that MET events are initiated due to the lack of EMT-inducing signals at attachment sites of metastatic tumor cells. In support, numerous examples of advanced carcinomas exist, showing that mesenchymal cells can regain characteristics of epithelial cells or undergo MET (104). It is now generally accepted that the reverting to an epithelial phenotype through MET represents a protective mechanism against host-immune attacks and creates resistance to anti-cancer drugs. The transdifferentiation into an epithelial phenotype and the formation of tight junctions between malignant cells that prevent penetration of host anti-tumor immune cells, host anti-tumor antibodies, and therapeutics represents one of the most basic cancer resistance mechanisms.

Importantly, changes between EMT and MET occur gradually, which leads to a wide range of intermediate cell stages that consequently possess an E/M hybrid phenotype. The E/M hybrid phenotype is especially prominent in the invasive front of several carcinomas where it has also initially been linked to cells with a stem cell-like phenotype (112, 113). We have recently shown in cancer cells derived from ovarian cancer biopsies that CSCs generate mesenchymal cells via EMT *in vitro* and undergo MET to form tumors containing epithelial cells when injected into immunodeficient mice (135). A marker combination widely used to identify CSCs in multiple cancers including prostate, pancreatic, and colon is EpCAM and CD44 (136, 137). Interestingly, the expression of these proteins can be accredited to epithelial and mesenchymal cells, respectively, suggesting a more general pattern of an E/M hybrid phenotype for tumor-initiating cells (TICs) (25). Very recently, work by Yu et al. demonstrated that cells, which leave the primary tumor, possess an epithelial or E/M hybrid phenotype. In the bloodstream these circulating tumor cells are bound by platelets, which trigger EMT via TGF-β signaling (138). However, cells undergoing EMT that leave the primary tumor experience a proliferation arrest, which is mediated by EMT inducers, like Twist1. In order to reenter a proliferative stage that allows for colonization and macrometastasis, the downregulation of Twist1 and concomitant MET is critically needed, while ongoing EMT signaling leads to dormancy and micrometastases at sites of reattachment (139). Additionally, it was recently reported that two distinct types of EMT exist in carcinomas, depending on the presence or absence of EMT inducer paired-related homeobox transcription factor 1 (Prrx1). When Prrx1 is expressed in cells undergoing Twist1-induced EMT, a CSC pattern is suppressed and cells fail to colonize. After Prrx1 is downregulated and other EMT inducers, such as Twist1 or ZEB1 have vanished, MET occurs and metastatic growth can be initiated (140). Notably, MET has also been reported in non-epithelial cancers, e.g., sarcoma (141, 142).

## Strategies to Degrade Tumor ECM Proteins or Downregulate Their Expression

Tumor-derived ECM plays an important role in inhibiting penetration and dispersion of cancer therapeutic agents within tumor masses and has been implicated in resistance to therapy of solid tumors (143). This has been shown for therapeutic modalities such as oncolytic Ads (21), antibodies (18, 19), immunotoxins (24), interferons (23), or complement (144). A series of approaches have been tested to partially degrade ECM proteins and improve the penetration of macromolecules and nanoparticle-based drugs. (i) The first type of approaches involves the intratumoral injection of proteases that can target ECM proteins. These proteases include trypsin, collagenase, hyaluronidase, MMPs, relaxin, and decorin. For example, intratumoral injection of collagenase has been shown to remove diffusive hindrance to the penetration of therapeutic molecules in subcutaneous human osteosarcoma and glioblastoma multiforme xenografts (145, 146). Similar approaches have been tested in combination with cancer virotherapy, including Ads (diameter: ~100 nm) and herpes simplex virus (HSV) (diameter: ~190 nm). These viruses represent prototypes of nano-particles and lessons learned from studies with oncolytic viruses are relevant for other large anti-cancer drugs. Direct injection of subcutaneous human glioblastoma multiforme tumor with a proteolytic enzyme (trypsin) or a protease mixture (collagenase/dispase) before intratumoral injection with reporter gene-expressing Ad vector elicited enhanced virus-mediated gene expression within the solid tumor (147). Intratumoral co-injection of collagenase with an oncolytic HSV vector in a human melanoma xenograft resulted in increased intratumoral viral spread and therapeutic benefit (148). Likewise, co-delivery of hyaluronidase and oncolytic Ads led to improved intratumoral diffusion and virus potency through degradation of hyaluronan-rich ECM in human prostate and melanoma xenograft models (149). (ii) The second approach involves the delivery of a protease-encoding gene expression cassette to tumors. Cheng et al. generated replication-incompetent Ads expressing MMP-8 that breaks down collagen type I, II, and III in subcutaneous human A549 lung cancer and BxPC-3 pancreatic cancer xenograft tumors (150). In studies testing MMP-8-expressing Ads, MMP-8 expression efficiently degraded collagen *in vitro*. Furthermore, co-injection of MMP-8-expressing Ads in combination with wild-type Ads resulted in reduced tumor cell growth and collagen expression within areas of virus-induced necrosis compared with wild-type Ad given together with a control Ad vector. Moreover, Mok et al. showed that intratumoral expression of MMP-1 and MMP-8 in the human HSTS26T soft tissue sarcoma xenograft degraded collagen, reduced the levels of sulfated proteoglycans, and increased spread and effectiveness of an oncolytic HSV (151). While degradation of ECM with enzymes, such as collagenase and MMPs, may improve viral penetration and distribution, there is a concern that this strategy may also increase tumor spread; MMPs and collagenase play an important role in tumor invasion and metastasis, which might limit the use of these proteins in a clinical setting (152, 153). Therefore, further thorough and detailed studies are required to gain an improved understanding of the potential risk associated with combined replicating oncolytic virus and ECM-degrading enzyme or protein therapy.

Our laboratories have tested approaches involving the expression of relaxin (154–157) or decorin (158). We will therefore describe these approaches in more detail.

### Relaxin-based approaches to increase drug penetration in solid tumors

**Figure 3:**
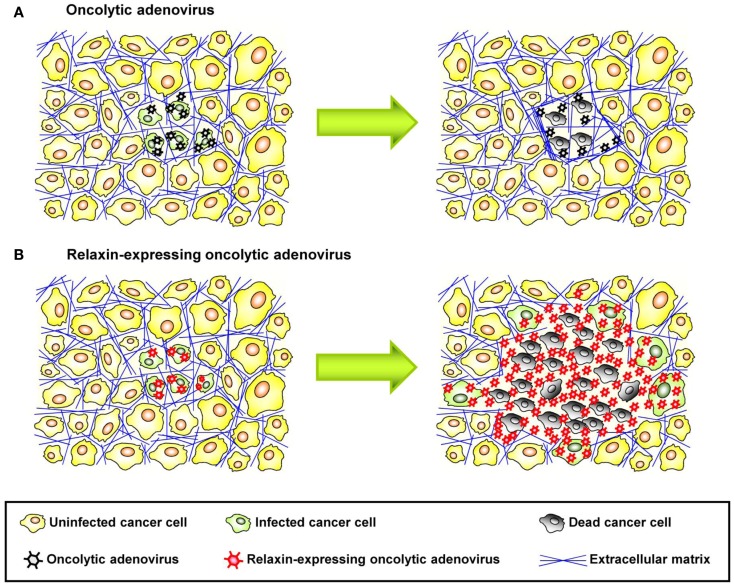
**Therapeutic effect of relaxin-expressing oncolytic adenovirus**. **(A)** ECM acts as a physical barrier in solid tumors, so that interstitial viral penetration and cell-to-cell spread of conventional oncolytic adenoviruses is restricted to the site of administration, leading to limited therapeutic efficacy. **(B)** Relaxin-expressing oncolytic adenovirus decreases ECM components within a tumor mass and increases its tumor penetration and dispersion, thereby eliciting improved antitumor efficacy.

Relaxin is an insulin-related peptide hormone (159). During pregnancy, relaxin has an integral role in softening the uterine cervix, vagina, and interpubic ligaments in preparation for parturition (160). Relaxin is the ligand for two leucine-rich repeat-containing G protein coupled receptors (LGRs), LGR7, and LGR8, now classified as relaxin family peptide receptors 1 and 2 (RXFP1 and RXFP2), respectively (161). These receptors have been found on relaxin target tissues, particularly on endometrial stromal cells and CAFs (161). Binding of relaxin to these receptors triggers intracellular signaling resulting in downregulation of ECM protein expression and upregulation of MMPs, which degrade stroma proteins. Importantly, relaxin decreases the synthesis of collagens and increases the expression of MMPs when collagen is abnormally upregulated, but it does not significantly alter basal levels of collagen expression, in contrast to other collagen-modulatory cytokines (e.g., interferon-γ) (162). This implies that relaxin acts predominantly on tissues with increased ECM protein expression such as fibrotic tissues and tumors. In agreement with these observations, earlier reports showed that relaxin expression mediated by an Ad vector reversed cardiac fibrosis without adversely affecting normal collagen levels in other organs in a transgenic murine model of cardiac fibrosis (163). Relaxin has been used for degradation of tumor-derived ECM components (164). In immunodeficient mice bearing human HSTS26T soft tissue sarcomas in dorsal skinfold chamber, chronic relaxin treatment via osmotic pumps elicited improved collagen down-regulation and intratumoral dispersion of macromolecules in tumor tissues, whereby the new tumor ECM, that is generated after relaxin treatment, was more porous and had a decreased diffusive resistance (145). *In vitro* studies with human OHS osteosarcoma multicell spheroids, recombinant relaxin increased the diffusion of the 150 kDa FITC-dextran, in part, due to increased production of collagenase (146). Similar results were reported in *in vivo* tumor models after intratumoral injection of recombinant relaxin (145). In cancer virotherapy studies, relaxin was demonstrated to enhance tumor penetration and dispersion of oncolytic Ad, thereby eliciting improved cancer gene therapy (155) (Figure 3). In this study, the growth of both subcutaneous xenograft (human glioma, hepatocellular carcinoma, cervical carcinoma, and lung carcinoma) and orthotopic tumors (human hepatocellular carcinoma) treated with relaxin-expressing oncolytic Ad was markedly inhibited compared to tumors treated with control oncolytic Ad that did not express relaxin. When viral persistence and distribution was confirmed by immunohistochemical studies, more Ad particles were observed across wider areas of tumor tissues treated with relaxin-expressing oncolytic Ad. Moreover, the collagen content of tumor tissues was reduced significantly by relaxin-expressing oncolytic Ad without affecting adjacent normal tissue. A relaxin-expressing oncolytic Ad containing Ad5/35 chimeric fibers in a subcutaneous human A375-mln1 malignant melanoma xenograft model also exhibited increased viral spread and transduction efficiency through the tumor mass and thereby increased anti-tumor efficacy and overall survival in metastatic tumor models (154).

Access of anti-tumor immune cells and their intratumoral infiltration is limited by tumor stroma (19). More specifically, the tumor stroma contributes to tumor immune escape by creating a physical barrier formed by ECM components that restricts direct physical contact between tumor-infiltrating anti-tumor immune cells and cancer cells. As an approach to overcome this limitation, Li et al. showed that the inducible intratumoral expression of relaxin through the transplantation of mouse hematopoietic stem cells transduced with a relaxin-expressing lentivirus vector led to suppressed tumor growth in an immunocompetent mouse breast cancer model (157). The therapeutic mechanism of the anti-tumor effect is associated with the degradation of tumor stroma mediated by relaxin and enhanced anti-tumor immune responses mediated by better intratumoral infiltration of anti-tumor immune cells. The same investigators also tested whether intratumoral relaxin expression facilitates transplanted anti-tumor T cells to control tumor growth. In a breast cancer model, they demonstrated that relaxin augmented the efficacy of *neu*-targeted adoptively transferred T cells, and improved survival of mice with *neu*-expressing mammary tumors. At day 33, in the T cell transplanted group, 25% of the mice were alive. Combined with relaxin expression, survival increased to 62.5%. Relaxin expression combined with naïve T cell treatment also increased survival (37.5%), compared to naïve T cell treatment alone (0%). Better survival of relaxin-expressing mice was due to a higher number of *neu*-specific T cells inside the tumor.

Tumor ECM as well as tumor cell density can inhibit diffusional transport of monoclonal antibody therapeutics in tumor tissues. In a study of the monoclonal antibody trastuzumab (Herceptin) penetration, Beyer et al. observed extensive tumor ECM and intercellular junctions in breast cancer patients and xenograft models (156). Therefore, the authors hypothesized that this hinders the access to Her2/neu and/or the intratumoral dispersion of trastuzumab. They showed that hematopoietic stem cell-mediated intratumoral relaxin expression in combination with trastuzumab therapy resulted in a decrease of ECM proteins and a significant delay of tumor growth, indicating that a stem cell-based approach for relaxin expression in tumors facilitates tumor ECM degradation and substantially enhances effectiveness of antibody therapy of cancer.

In all of these studies, relaxin expression did not induce metastasis. In fact, it reversed the spread of tumor cells that normally would metastasize. The latter is in conflict with earlier studies by Silvertown et al. reporting that permanent relaxin overexpression increased *in vivo* prostate xenograft tumor growth and angiogenesis (165). These results were recently revised by the same group (166). While short-term exposure of tumor cells to relaxin *in vitro* seems to enhance invasiveness (167), long-term exposure reduces it (168). The general consensus is that relaxin expression alone is not sufficient to induce metastasis, a process that involves dissociation of cells from the primary tumor, enhanced cell motility, and the ability of cells to invade blood vessels and to grow effectively at distant sites (154).

### Decorin-based approaches to increase drug penetration in solid tumors

Decorin, a small leucine-rich proteoglycan consisting of a core protein and a single glycosaminoglycan chain, is a ubiquitous component of ECM. Decorin has an impact on the production of several ECM components. For example, it regulates collagen fibril formation by interacting with collagen fibrils and delaying the lateral assembly of individual triple helical collagen molecules, leading to the reduced diameter of the fibrils (169). Decorin also influences the production of other ECM components by inhibiting the expression of TGF-β, a key profibrotic growth factor (170). Moreover, decorin has an important role in inducing ECM remodeling through promotion of MMP-1 activity (171). These observations suggest that decorin can modulate tumor ECM production and composition at several levels, and hence has an integral role in degradation and/or downregulation of tumor ECM constituents. Downregulation of TGF-β production by decorin could also facilitate anti-tumor immune responses through inhibition of immuno-suppressive T cells (172). In an oncolytic virotherapy study using decorin, Choi et al. showed that tumor tissue dispersion by decorin-expressing oncolytic Ad was substantially enhanced compared with that of control oncolytic Ad, in tumor spheroids prepared from glioma or breast cancer patients as well as established subcutaneous human glioma xenograft tumors *in vivo* (158). In this study, decorin-expressing oncolytic Ad significantly reduced ECM components within the tumor tissues while normal tissue adjacent to the tumor was not affected. Decorin-expressing oncolytic Ad therefore led to dramatically increased anti-tumor effect as well as survival benefit in a variety of tumor xenograft models. Importantly, intratumoral administration of decorin-expressing oncolytic Ad to the primary tumor site substantially reduced the formation of B16BL6 melanoma pulmonary metastases in mice, indicating that this approach is capable of inducing a systemic anti-tumor immune response (158).

## Strategies to Open Epithelial Junctions

### Junction openers

Various pathogens must first breach the epithelial barrier before gaining access to the body in order to initiate infection. Several mechanisms to disrupt junctional integrity developed in these pathogens, e.g., *Clostridium perfringens* enterotoxin removes claudins-3 and -4 from tight junctions to facilitate bacterial invasion (173). Also, *zona occludens* toxin (Zot) is produced by *Vibrio cholerae* strains and possesses the ability to reversibly modify intestinal epithelial tight junctions, granting the passage of macromolecules through mucosal barriers (174). Notably Cox et al. have shown that Zot increases the transport of drugs with low bioavailability (e.g., paclitaxel, doxorubicin, acyclovir, and cyclosporin A) up to 30-fold (175). Additionally, oncoproteins encoded by human papillomavirus (HPV), human Ad, and human T-lymphotropic virus 1 (HTLV-1) can transiently open tight junctions by the mislocalization of the tight junction protein ZO-1, thereby enhancing paracellular permeability in epithelial cells (176). It is intriguing that several viruses target epithelial junction protein to achieve infection of and dissemination in epithelial tissues. Most species of human adenoviruses (except species B) binds to the CAR. CAR is a tight junction protein. A number of studies have demonstrated that during replication of Ad5, excess production of fiber or fiber/penton base complexes results in the disruption of epithelial junctions either by interfering with CAR dimerization or by triggering intracellular signaling that leads to reorganization of intercellular junctions (177, 178). Measles virus uses the adherence junction protein nectin 4 (179). Finally, we have shown that species B adenoviruses target the desmosomal junction protein DSG2. To date, however, there are no epithelial junction openers that are being used for cancer therapy. A number of chemical detergents, surfactants, calcium-chelating agents, and phospholipids have been used to increase drug absorption through the gastrointestinal (GI) tract epithelium (180). Recently, Kytogenics Pharmaceuticals, Inc., has developed a tight junction opener based on chitosan derivatives. It is thought to act by electronegative forces applied to tight junction proteins. However, all of these agents act indiscriminately to mechanically disrupt junctions and cannot be applied systemically without major toxic side effects.

### Ad serotype 3 derived junction opener JO-1

**Figure 4:**
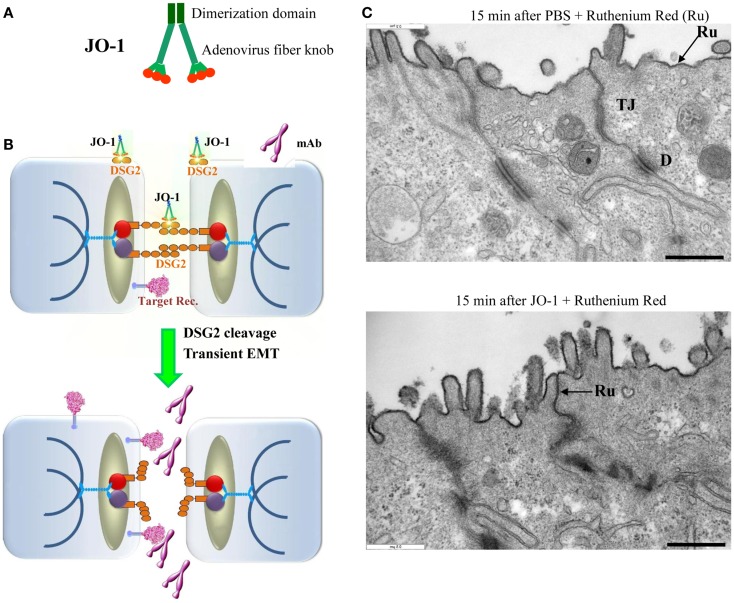
**Junction opener 1 (JO-1)**. **(A)** Schematic structure of JO-1 The Ad serotype 3 fiber knob domain and one fiber shaft motif was fused through a flexible linker to a homodimerizing K-coil domain (182). The protein is self-dimerizing and can be purified by His-Ni-NTA affinity chromatography. **(B)** Mode of action. JO-1 binds with picomolar avidity to DSG2. In epithelial cancer cells, DSG2 is overexpressed and exposed on the cell surface with preferential localization to desmosomes. JO-1 binding to DSG2 triggers cleavage of DSG2 dimers between neighboring cells and the transient activation of EMT pathways. This triggers junction opening and relocalization of target receptors that are often trapped in epithelial junctions. Junction opening allows for access of drugs (for example mAbs) to their target receptors. **(C)** Transmission electron microscopy of junctional areas of T84 cells. Cells were either treated with PBS (upper panel) or JO-1 (lower panel) for 1 h on ice, washed, and then incubated for 15 min at 37°C. At this time, the electron-dense dye ruthenium red (Ru) (1) was added together with the fixative. If tight junctions (above the desmosomes) are closed, the dye only stains the apical membrane (black line). If tight junctions are open, the dye penetrates between the cells and stains the baso-lateral membrane. JO-1 also mediates the partial dissociation of desmosomes (D). The scale bar is 0.5 μm.

Human Ads have been classified into 7 species (A to G) currently containing 57 serotypes. Wang et al. recently reported that a group of human Ads uses DSG2 as a receptor for infection (181). Among DSG2-targeting viruses is serotype 3 (Ad3). Ad3 is able to efficiently breach the epithelial barrier in the airway tract and infect airway epithelial cells. This is achieved by the binding of Ad3 to DSG2, and subsequent intracellular signaling that results in transient opening of tight junctions between epithelial cells. Wang et al. have capitalized on this mechanism and created a recombinant protein that contains the minimal structural domains from Ad3 that are required for opening of the intercellular junctions in epithelial tumors. This protein is called “junction opener 1” or “JO-1.” JO-1 is a self-dimerizing recombinant protein derived from the Ad3 fiber (182). JO-1 has a molecular weight of approximately 60 kDa (Figure 4A). It can be easily produced in *E. coli* and purified by affinity chromatography. JO-1 binding to and clustering of DSG2 triggers EMT that results in transient opening of epithelial junctions, in polarized epithelial cancer cells *in vitro* (Figures 4B,C) and *in vivo*, in mouse models with epithelial tumors. Wang et al. have shown in over 25 xenograft tumor models that the intravenous injection of JO-1 increased the efficacy of cancer therapies, including many different monoclonal antibodies and chemotherapy drugs, in a broad range of epithelial tumors. Further studies showed that the effective doses of chemotherapy can be reduced when the chemotherapy drugs are combined with JO-1. Finally, studies have demonstrated that combining JO-1 with chemotherapy drugs markedly reduced the toxic side effects of chemotherapy. The application of JO-1 was safe and well-tolerated in toxicology studies carried out in human DSG2-transgenic mice and macaques (109, 181).

#### Mechanism of JO-1-mediated junction opening

Wang et al. suggested that at least two mechanisms are involved in JO-1-mediated opening of tight junctions: DSG2 cleavage/internalization and EMT-like intracellular signaling. Epithelial cells are linked to each other by homodimers of DSG2. Studies *in vitro* and in xenograft models have shown that the DSG2 ECD is cleaved upon binding of JO-1, which results in DSG2 internalization (109). On the other hand, a series of data indicate that Ad3 binding triggers EMT-like signaling, which most likely involves the intracellular domain (ICD) of DSG2. Using mRNA expression arrays and qRT-PCR, Wang et al. found 12 h after incubation of polarized breast cancer epithelial cells with a JO-1-like ligand that 430 genes were upregulated and 352 genes were downregulated compared to incubation with a control protein (181). mRNA expression profiling revealed the activation of pathways involved in EMT (MAPK/ERK, adherens junctions, focal adhesion, and regulation of actin cytoskeleton signaling). Further studies showed an increase in PI3K and MAPK/ERK1/2 phosphorylation within 1 h after incubation with Ad type 3 pento-dodecahedral particles or JO-1 (109, 181). PI3K and MAPK/ERK1/2 activation was significantly decreased in cells in which DSG2 expression was suppressed by siRNA. Beyer et al. also found that subsequently to MAPK and PI3K activation, the protein levels of E-cadherin, a key junction protein, decreased in epithelial cells, indicating a down-regulation of gene expression of junction proteins (109).

#### JO-1 increases the efficacy of cancer therapy by monoclonal antibodies and chemotherapy drugs

**Figure 5:**
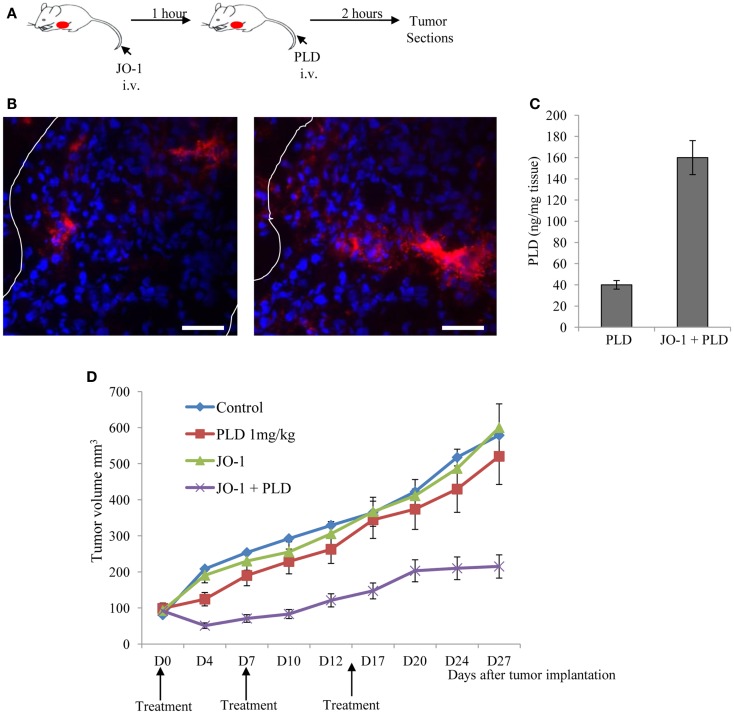
**Junction opener 1 increases tumor penetration and efficacy of PEGylated liposomal doxorubicin (PLD)/Doxil™**. Studies were performed in mice with mammary fat pad tumors derived from ovc316 cells (135, 206). Ovc316 cells are Her2/neu positive epithelial tumor cells derived from an ovarian cancer biopsy. **(A)** Scheme of experiment on tumor penetration of PLD. Mice were intravenously injected with PBS or JO-1 (2 mg/kg) followed by PLD or PBS 1 h later. Two hours after PBS or PLD injection, mice were sacrificed and tumors harvested. **(B)** Immunofluorescence analysis for PLD on tumor sections with anti-PEG antibodies. PLT appears in red. The scale bar is 20 μm. Notably, free PEG is poorly detected by ELISA or immunohistochemistry (184). **(C)** PLD concentrations in tumors measured by ELISA (*N* = 3). **(D)** Therapy study in mice with ovc316 tumors. Treatment was started when tumors reached a volume of 100 mm^3^ (D0). Mice were injected intravenously with 2 mg/kg JO-1 or PBS, followed by an intravenous injection of PLD (1 mg/kg) or PBS 1 h later. Treatment was repeated weekly (*N* = 5).

Beyer et al. have also shown in mouse xenograft tumor models that the i.v. administration of JO-1-mediated junction opening in epithelial tumors (183). The changes triggered by JO-1 were detectable within 1 h after its i.v. injection. This, subsequently, enabled the increased intratumoral penetration of the anti-Her2/*neu* mAb trastuzumab (109). These biological effects of JO-1 translated into an increased therapeutic efficacy of several mAbs, including trastuzumab and cetuximab, in xenograft tumor models, e.g., models of colon, breast, gastric, lung, and ovarian cancer (109). JO-1 co-administration also enhanced the therapeutic efficacy of several chemotherapy drugs, including PEGylated liposomal doxorubicin (PLD or Doxil^®^), paclitaxel (Taxol^®^), nanoparticle albumin bound paclitaxel (Abraxane^®^), and irinotecan (Camptosar^®^) in tumor xenograft models of breast, lung, and prostate cancer (183). Furthermore, chemotherapy doses could be decreased without compromising the anti-tumor effects due to JO-1 co-therapy. This also provided protective effects to normal tissues (183). For example, we showed that the ability of JO-1 to open intercellular junctions in tumors increased the uptake and amount of chemotherapeutics in the tumor environment (Figures 5A–C). This then resulted in reduced drug levels in normal tissues, thereby providing a larger therapeutic window. Immunofluorescence analysis of tissue sections also revealed higher levels of PLD in tumors of JO-1 + PLD treated mice compared to mice treated with PLD alone. In these animals, PLD is found to be more broadly distributed over a greater distance from blood vessels, suggesting better intratumoral penetration and absorption by tumor tissue (Figure 5B). Using an ELISA to measure PEGylated compounds in tissues (184), we found more PLD in tumors and less in normal tissues of mice that received JO-1 prior to i.v. PLD injection (Figure 5C). Better intratumoral penetration and accumulation of PLD after JO-1-mediated junction opening, resulted in enhanced therapeutic efficacy of PLD chemotherapy. This was shown in a model with mammary fat pad tumors derived from primary ovarian cancer cells (obtained from a patient biopsy) (135). This cell line was nearly resistant to PLD injected intravenously at a dose that corresponds to PLD doses used in patients. Importantly, JO-1 pre-treatment significantly improved PLD therapy (Figure 5D). JO-1 also relieved adverse side effects from PLD treatment, e.g., liver enzymes (AST, ALT, and alkaline phosphatase) were significantly decreased in animals treated with JO-1 and PLD compared to mice treated with PLD alone. Mice that received JO-1 injections also had less severe tissue damage in the bone marrow and intestine caused by PLD treatment (183).

#### Relocalization of target receptors

In breast cancer xenograft sections and in cultured breast cancer cells, Beyer et al. found co-staining of Her2/*neu* and the adherens junction protein claudin 7 (183). Confocal microscopy of breast cancer BT474 cells confirmed the trapping of Her2/*neu* in lateral junctions. Incubation of the Her2/*neu* positive breast cancer cell lines BT474 or HCC1954 with JO-1 changed the composition of the lateral epithelial junctions within 1 h. As a result of this, Her2/*neu* staining at the cells surface became more intense, while it faded in areas distal of the cell surface. This suggests that JO-1-mediated junction opening triggered a translocation of Her2/*neu* from lateral membranes to the cell surface.

#### Tumor-specificity of JO-1 action

Junction opener 1 does not efficiently bind to mouse (185), hamster, or dog DSG2 (André Lieber, unpublished data). To perform efficacy and safety studies in a small animal model, we generated human DSG2-transgenic mice that expressed human DSG2 at a level and in a pattern similar to humans (185). Using the hDSG2-transgenic mouse model with syngeneic hDSG2^high^ tumors, we demonstrated that JO-1 predominantly accumulates in tumors (183). This could be explained by either one of the following factors: (i) the overexpression of DSG2 by tumor cells, (ii) better accessibility of DSG2 on tumor cells due to a lack of strict cell polarization compared to DSG2-expressing normal epithelial cells, or (iii) a high degree of vascularization and vascular permeability in tumors.

#### Toxic side effects and immunogenicity

The i.v. injection of JO-1 at a dose of 2 mg/kg into hDSG2-transgenic mice had no observed adverse side effects, except for mild, transient diarrhea. There were also no abnormalities found in laboratory parameters as well as histopathological studies of tissues. We speculate that this is due to the fact that DSG2 in tissues, other than the tumor and a subset of epithelial cells in the intestine/colon, is not accessible to i.v. injected JO-1. The hDSG2-transgenic mouse model was also used to obtain biodistribution and pharmacokinetics data for JO-1 (183).

Recently, we started safety studies with JO-1 in macaques. So far, we injected two animals intravenously with JO-1 at a dose of 0.6 mg/kg and performed a full necropsy 3 days later. Behavior and health was normal in both animals. In blood and tissue analyses, we did not find hematological or histological abnormalities, except for a mild inflammation in the small intestine. JO-1 binding to DSG2 on tumor cells triggers pathways involved in EMT, a process which, as mentioned above, has been associated with tumor metastasis. Over 20 *in vivo* studies conducted with JO-1 combined with a range of cancer therapeutics in various different cancers with long-term follow-up, have not provided any evidence of metastases (183). Transient activation of the EMT pathway is only one of many steps required for tumor metastasis. Detachment of tumor cells from epithelial cancers and their subsequent migration is only possible after long-term crosstalk between malignant cells and the tumor microenvironment, resulting in changes in the tumor stroma and phenotypic reprograming of epithelial cells into mesenchymal cells (186).

Junction opener 1 is a protein derived form Ad3 and therefore potentially immunogenic. This might not be a critical issue if JO-1 is used in combination with chemotherapy, which suppresses immune responses (187–189). In addition, Beyer et al. have shown that JO-1 remains active *in vitro* and *in vivo*, even in the presence of anti-JO-1 antibodies generated by the JO-1 vaccination of mice (183). This may be due to the fact that JO-1 binds to DSG2 with a very high avidity, thus potentially disrupting the complexes between JO-1 and antibodies against JO-1. Notably, JO-1 is a dimer of a trimeric fiber knob, which contributes to the picomolar avidity to DSG2 (182). Wang et al. performed repeated injections of JO-1 in an immunocompetent hDSG2 mouse tumor model to test the effect of anti-JO-1 antibodies on the therapeutic efficacy of JO-1 (185). Importantly JO-1 had an enhancing effect on PLD therapy after repeated JO-1 pre-treatment, demonstrating that JO-1 continues to be effective after multiple treatment cycles, even in the presence of detectable antibodies.

## Strategies to Remodel the Tumor Stroma through Targeting of TAMs

As outlined in chapter 1.2, bone marrow-derived cells, including TAMs, have a pro-tumor effect, in part through the stimulation of ECM protein synthesis, which in turn blocks intratumoral penetration of drugs. Therefore, killing of TAMs should, theoretically, increase the intratumoral penetration and accumulation of anti-cancer drugs. TAM depletion results in tumor growth suppression. This has been shown in animal models of cancer by using transgenic mice (190, 191), clodronate liposome-depletion of macrophages (192), DNA vaccination against macrophages (193), and neutralizing antibodies against macrophage chemoattractants (194). For example, Zeisberger et al. showed that combining macrophage depletion with antibody therapy greatly decreased the tumor size (195). Furthermore, a recent study showed that TAM targeting by inhibiting either the myeloid cell receptors colony stimulating factor-1 receptor (CSF1R) or chemokine (C–C motif) receptor 2 (CCR2) decreased the number of TICs in pancreatic tumors, improved chemotherapeutic efficacy, inhibited metastasis, and increased anti-tumor T cell responses (196). Overall these studies showed that decreasing the number of TAMs in the tumor stroma effectively altered the tumor microenvironment and markedly suppressed tumor growth and metastasis. Targeting TAMs did not interfere with the biological functions of M1 macrophages, including cytotoxicity and antigen presentation.

We are currently attempting to deliver a suicide gene to TAMs using TAM targeting Ad vectors or the HSC-based approach described for relaxin gene delivery (157). To restrict suicide gene expression to TAMs we utilized a miRNA-based system that avoids transgene expression in other myeloid cells. As a therapeutic gene for killing TAMs, we are currently focusing on the enzyme cytosine deaminase, which converts the prodrug 5-Flurocytosine (5-FC) to the active chemotherapeutic agent 5-Fluorouracil (5-FU) (197). Toxic 5-FU and metabolites diffuse out of TAMs to surrounding cells killing TAMs as well as neighboring dividing tumor cells.

## Strategies to Inhibit EMT to Remodel the Tumor Stroma

As outlined above, EMT in solid tumors promotes the expression of several ECM proteins and thus blocks penetration of anti-cancer drugs. This give a rationale for inhibiting EMT processes in epithelial tumors. Accumulating knowledge about EMT pathways in solid tumors led to the development of EMT targeted therapies (198). Classically, such treatment strategies concentrate on the blockage of ATP-binding sites in affected kinases using small molecule inhibitors, as the RTK inhibitor Gefitinib for treatment of non-small cell lung cancer with activated EGFR mutation (199). While originally designed for their anti-proliferative effect on cancer cells, it was also shown that such molecules can influence the EMT status. In a cell-based screening of 267 small molecules, several compounds targeting ALK5, MEK, and SRC kinases were identified as potent inhibitors of EMT induced by EGF, HGF, and IGF-1 (200). It was also shown that counteracting TGF-β-induced EMT by treatment with troglitazone or knockdown of Smad3 in tumor cells can significantly inhibit experimental metastasis in mice (201). Furthermore, targeting early specific EMT events, like the degradation of epithelial basement membranes, can be a successful strategy, as shown in renal interstitial fibrosis. Deficiency of plasminogen activator (tPA), which is a potent activator of MMP-9, resulted in stable epithelial basement membranes and inhibition of EMT (202). Furthermore, studies involving the expression of pro-epithelial factors such as BMP-7 and Dkk, have demonstrated the inhibition of EMT and consequent CSC induction as well as metastasis in colon and prostate cancer models (203, 204). In pioneering work to understand the complexity underlying EMT induction, Scheel and colleagues showed recently that canonical and non-canonical Wnt signaling cooperate with TGF-β in order to initiate EMT in breast cancer (205). Consequently, a series of commercial or experimental Wnt-pathway inhibitors counteracted EMT. In summary, similar pathways promote therapy resistance, EMT, CSCs, and metastasis. This gives a rationale to inhibit processes that induce the mesenchymal phenotype of cancer cells. Clearly, inhibition of EMT must target a variety of pathways and should only be considered when combined therapy is applied that targets proliferating cells.

## Conclusion

Most solid tumors are derived from epithelial cells. Malignant tumor cells actively protect themselves from host-immune responses and anti-cancer therapeutics by creating physical barriers that prevent the intratumoral penetration and contact to malignant cells. This is achieved by the production of cytokines and chemokines that attract fibroblasts and myeloid cells into the tumor and differentiate them into cells that support tumor growth and produce ECM proteins that shield nests of malignant tumor cells. Furthermore, although malignant cells display a high degree of dedifferentiation, they maintain epithelial junctions that seal the paracellular space between tumor cells and block access to tumor antigens or target receptors. Tumor ECM and epithelial junctions represent the most basic mechanisms that create resistance to cancer treatment. Because of their importance to the tumor, they also represent an “Achilles’ heel” that can be used for cancer therapy. Removal of these barriers will either directly negatively affect tumor cells or facilitate anti-tumor immune responses and drug treatment, through better intratumoral penetration and accessibility of target cells. A number of experimental approaches are aimed toward the transient degradation or downregulation of ECM proteins using injection of ECM-degrading enzymes into the tumor or their intratumoral expression after viral- or stem cell-based gene transfer. We recently finished a phase I clinical trial with a relaxin-expressing oncolytic Ad in patients with recurrent cancer, demonstrating a clinical benefit with a good safety profile. Furthermore, we are focusing on the clinical development of a recombinant epithelial junction opener to be used in combination with Doxil chemotherapy in ovarian cancer patients. Other approaches to overcome physical barriers in tumors are at a less advanced stage. These approaches attempt to indirectly decrease tumor-associated ECM by killing tumor stromal cells that produce ECM proteins (e.g., tumor-associated fibroblasts or macrophages). ECM production and epithelial junctions can also be targeted through influencing signaling pathways in tumor cells, specifically pathways involved in the regulation of EMT/MET and hypoxia. In conclusion, there is an increasing arsenal of approaches that can be used to enhance the efficacy of more classical cancer therapeutics and overcome treatment resistance.

## Conflict of Interest Statement

The authors declare that the research was conducted in the absence of any commercial or financial relationships that could be construed as a potential conflict of interest.
